# Glutamatergic neurons in paraventricular nucleus of the thalamus regulate the recovery from isoflurane anesthesia

**DOI:** 10.1186/s12871-022-01799-y

**Published:** 2022-08-11

**Authors:** Xiaoli Bu, Yiqiu Chen, Ping Lv, Xiaoyun Fu, Bao Fu

**Affiliations:** 1 Department of Critical Care Medicine, Affiliated Hospital of Zunyi Medical University, Guizhou Province 563003 Zunyi city, China; 2 Guizhou Key Laboratory of Anesthesia and Organ Protection, Zunyi Medical University, Zunyi city, 563003 Guizhou Province China

**Keywords:** Paraventricular nucleus of the thalamus, Isoflurane, Emergence, Glutamate

## Abstract

**Objectives:**

Previous studies have demonstrated that the paraventricular nucleus of the thalamus (PVT) is a key wakefulness-controlling nucleus in the thalamus. Therefore, PVT may also be involved in the process of general anesthesia. This study intends to explore the role of PVT in isoflurane anesthesia.

**Methods:**

In the present study, we used the expression of c-Fos to observe the neuronal activity of PVT neurons under isoflurane anesthesia. We further recorded the effect of isoflurane anesthesia on the calcium signal of PVT glutamatergic neurons in real time with the help of calcium fiber photometry. We finally used chemogenetic technology to specifically regulate PVT glutamatergic neurons, and observed its effect on isoflurane anesthesia and cortical electroencephalography (EEG) in mice.

**Results:**

We found that glutamatergic neurons of PVT exhibited high activity during wakefulness and low activity during isoflurane anesthesia. Activation of PVT glutamatergic neuronal caused an acceleration in emergence from isoflurane anesthesia accompanied with a decrease in EEG delta power (1–4 Hz). Whereas suppression of PVT glutamatergic neurons induced a delay recovery of isoflurane anesthesia, without affecting anesthesia induction.

**Conclusions:**

Assuming a pharmacokinetic explanation for results can be excluded, these results demonstrate that the PVT is involved in regulating anesthesia emergence.

## Introduction

General anesthetics have been used clinically for more than 170 years, but the mechanism of reversibility leading to loss of consciousness has not been clarified. Previous studies have indicated that the transitions into both general anesthesia and natural sleep share some neuronal mechanisms [1–6]. Therefore, it seems feasible to explore the mechanism of general anesthesia from sleep arousal pathway.

The paraventricular nucleus of the thalamus (PVT) is an important node in the limbic system. Patients with impaired PVT showed disturbances of consciousness ranging from drowsiness to sleep-like coma [7]. A recent study has demonstrated that PVT is a key wakefulness-controlling nucleus in the thalamus [8]. PVT is thought to play a role in the loss of consciousness caused by general anesthetics. Therefore, this study used chemogenetic technology to explore the role of PVT in the process of consciousness loss caused by isoflurane.

## Materials and methods

### Animals

Our study was in accordance with the Guide for the Care and Use of Laboratory Animals in China (No. 14924, 2001) and was approved by the Animal Care and Use Committees of Zunyi Medical University. Adult male Vglut2-IRES-Cre mouse with C57BL/6 J background were purchased from Changsha Tianqin Technology Co., Ltd. (Changsha, China). Mice were raised in standard chambers within an SPF laboratory animal room (12/12-h light/dark cycle (light on at 6:00 am); 23 ± 2◦C; relative humidity: 55% ± 2%) with food and water adlibitum [9].

### Drugs

Isoflurane used in the study was obtained from RWD Life Science (Shenzhen, China). Lidocaine and pentobarbital were purchased from Chaohui Pharmaceutical (Shanghai, China). Clozapine N-oxide (CNO) was purchased from Sigma-Aldrich (United States, C0832).

### Virus injection

Mice were anaesthetized with 1.4% isoflurane with oxygen (O_2_) at 1 L/min. After anesthesia, the mouse head was secured in the brain stereotaxic frame. Then, the mouse head hair was shaved. Mouse skulls were exposed after lidocaine local anesthesia. Adeno-associated virus rAAV-hSyn-DIO-hM3Dq-mCherry, rAAV-Ef1-DIO-hM4Dq-mCherry and rAAV-hSyn-DIO-Gcamp6s were injected into PVT (coordinates, bregma: AP = -1.20 mm; ML =  + 0.55 mm; DV = -2.95 mm, angled at 10° towards the midline) through a glass micropipette by a microinjection pump (200 nl, 20 nl/min) [8]. For calcium fiber photometry, optic fibers were implanted over the PVT and secured with two skull screws and dental cement. Mice were allowed to recover for at least 3 weeks before electrophysiological or behavioral experiments. After experiments, histological analysis was performed to verify the location of viral transduction and fiber placements. Data was excluded from the analysis if viral transduction and fiber placements extended beyond the PVT region.

### Calcium fiber photometry recordings

A multichannel fiber photometry system (ThinkerTech Nanjing Bioscience Nanjing, China) equipped with a 480-nm excitation LED (3 W, CREE), a dichroic mirror (DCC3420M; Thorlabs) and a multifunction data acquisition software (Thinker Tech Nanjing Bioscience Inc.) were used to record the fluorescence signals of the GCaMP. Simultaneously filtered at 40 Hz and digitalized at 500 Hz. 3 weeks later, mice were used to record changes in GCaMP signals.

The GCaMP signals of mice during awake were recorded as the baseline. Then, the mice were anesthetized with 1.4% isoflurane for 10 min. The changes of GCaMP signals during anesthesia and recovery from general anesthesia were recorded continuously. Fiber photometry data were analyzed using MATLAB 2019a (MathWorks, Cambridge, United States). The values of fluorescence change (ΔF/F) were calculated using the following formula: (F—F_0_)/F_0_, where F is the test fluorescence signal and F0 is the basal signal.

### Behavioral tests

The loss of righting reflex (LORR) and recovery of righting reflex (RORR) are the traditional indicators of consciousness loss and recovery in rodents after general anaesthesia [10]. In the study, mice were placed into an anesthesia chamber (10 × 20 × 15 cm) for equilibrating 10 min. Subsequently, the mice were anesthetized by 1.4% isoflurane with 100% O2 at 1 l/min for 30 min. An anesthesia monitor (Vamos; Drager Company, Germany) was used to monitor the concentration of isoflurane in the chamber. During the experiment, the temperature of mice was maintained at 37.5℃. The duration from isoflurane inhalation to LORR was consider as the LORR time, while the duration from the end of isoflurane inhalation to RORR was defined as the RORR time.

For chemogenetic study, the mice in M3 and M4 group were intraperitoneal injected either CNO (1 mg/ml, 1 mg/kg, i.p.) or normal saline (NS, 0.9%, equal volume, i.p.) 1 h before the behavioral test and EEG recording. There was at least 1 week rest between CNO and saline in the same mouse. All mice were sacrificed after isoflurane anesthesia and subjected to immunofluorescence to verify the virus expression.

### EEG Recording and spectral analysis

EEG recording was synchronized with behavior test. Relative powers in the different frequency bands were computed by averaging the signal power across the frequency range of each band (δ: 1–4 Hz,: θ:4–8 Hz,α: 8–12 Hz, β: 12–25 Hz, and γ: 25–60 Hz) [9]. EEG signals were amplified by a model-3000 amplifier (A-M Systems, United States) and collected by a CED Power1401-3 device (Cambridge Electronic Design, Cambridge, United Kingdom).The signals were filtered between 0.1 and 300 Hz. Data were digitized and recorded using the Spike2 software package (Cambridge Electronic Design, Cambridge, United Kingdom) [10].

### Immunofluorescence

The mice were anesthetized by intraperitoneally injected with sodium pentobarbital (100 mg/kg). Then the mice were perfused with 0.1 M phosphate-bufered saline (PBS) followed by 4% paraformaldehyde in 0.1 M phosphate bufer through the left ventricle into the ascending aorta. The brains were collected and post-fixed in 30% sucrose at 4◦C until sank. The brains were sectioned into 30-mm slices on a cryostat (Leica CM1950). For the c-Fos experiment, we stained c-Fos in three groups (*n* = 8 for each group). In the anesthesia group, Vglut2-IRES-Cre mice were kept in an anesthesia state in the chamber with a constant level of isoflurane (1.4%) and oxygen (1 L/min) for 1.5 h. For the recovery group, mice were kept awake at room temperature for 1.5 h after administering isoflurane (1.4%) and oxygen (1 L/min) for 1.5 h. For the wake group, we stained c-Fos without operating. Firstly, the sections were incubated in a blocking buffer (PBS containing 2.5% normal goat serum, 1.5% bovine serum albumin and 0.1% TritonTM X-100) for 2 h at room temperature. Then the sections were incubated by the primary antibody (rabbit anti-cFos, 1:1000, Synaptic Systems) in the blocking buffer overnight at 4◦C, followed by a 3 min × 10 min wash with PBST (PBS with 0.1% Triton X-100, vol/vol). Subsequently, sections were incubated with the secondary Antibody (goat anti-rabbit conjugated to Alexa 594, 1:1000 dilution, Invitrogen) at room temperature for 2 h. Images of c-Fos immunostaining were captured on an Olympus BX63 virtual microscopy system [11].

### Statistical analysis

The data are expressed as mean ± SEM. The paired Student’s t-tests was used to compare the difference between the pre- and post-events in calcium signals. One-Way ANOVA were used to analysis the expression of c-Fos among three groups. The differences of LORR and RORR between the two groups were analyzed using unpaired Student’s t-tests. EEG changes in the power spectrum were analyzed by two-way ANOVA followed by a Bonferroni post hoc test. *P* < 0.05 was considered as significant.

## Results

### Effect of isoflurane anesthesia on the expression of c-Fos in PVT neurons

**Fig. 1:**
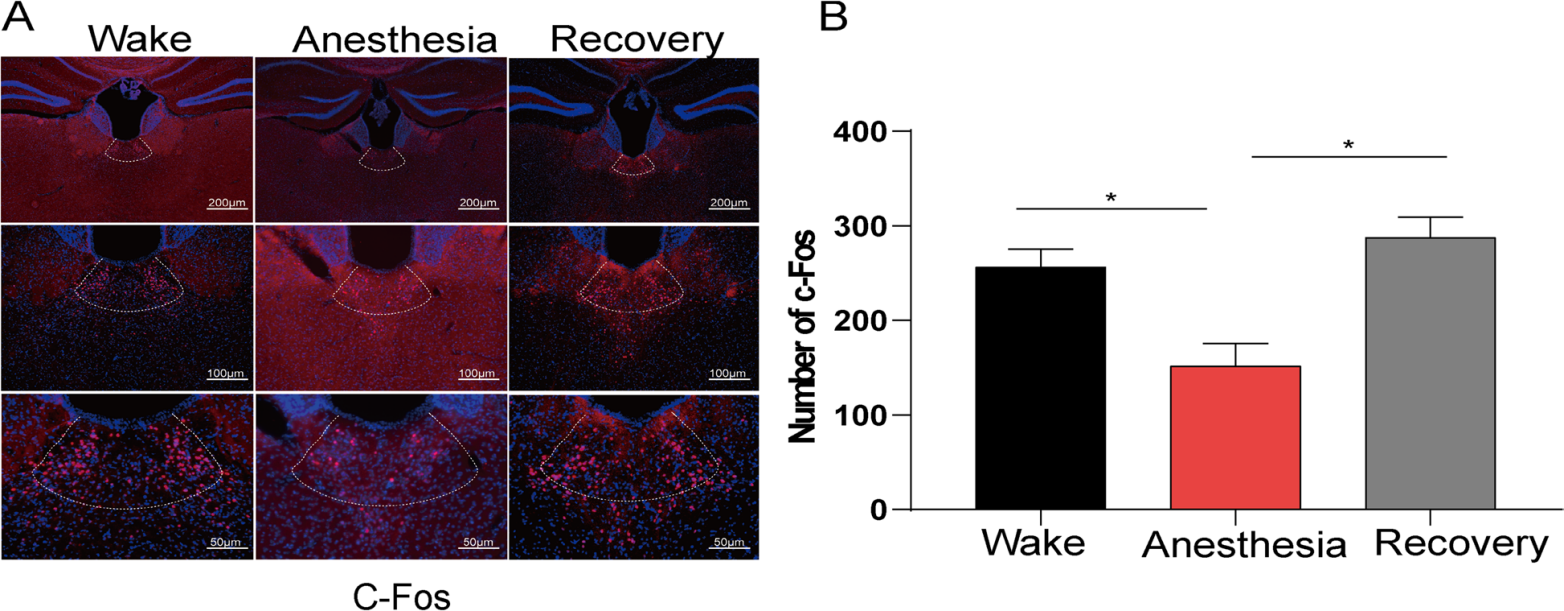
Expression of c-fos in paraventricular nucleus of the thalamus (PVT) neurons in mice under different conditions. **A** Immunofluorescence picture of c-fos expression. **B** The comparison of fluorescence intensity. ^*^P < 0.05, *n* = 8

We observed the effect of isoflurane anesthesia on the expression of c-fos protein in PVT neurons by immunofluorescence technique (Fig. 1A). There was no significant difference in the expression of c-Fos protein in PVT neurons between wake and recovery from isoflurane anesthesia (256.33 ± 19.22 *vs* 287.67 ± 21.67, *P* > 0.05). The expression of c-fos protein in anesthesia period was significantly lower than that in wake period and recovery period (151.67 ± 23.78 *vs* 256.33 ± 19.22; 151.67 ± 23.78 *vs* 287.67 ± 21.67, *P* < 0.01, Fig. 1B).

### Effect of isoflurane anesthesia on calcium signal of glutamatergic neurons in PVT

**Fig. 2:**
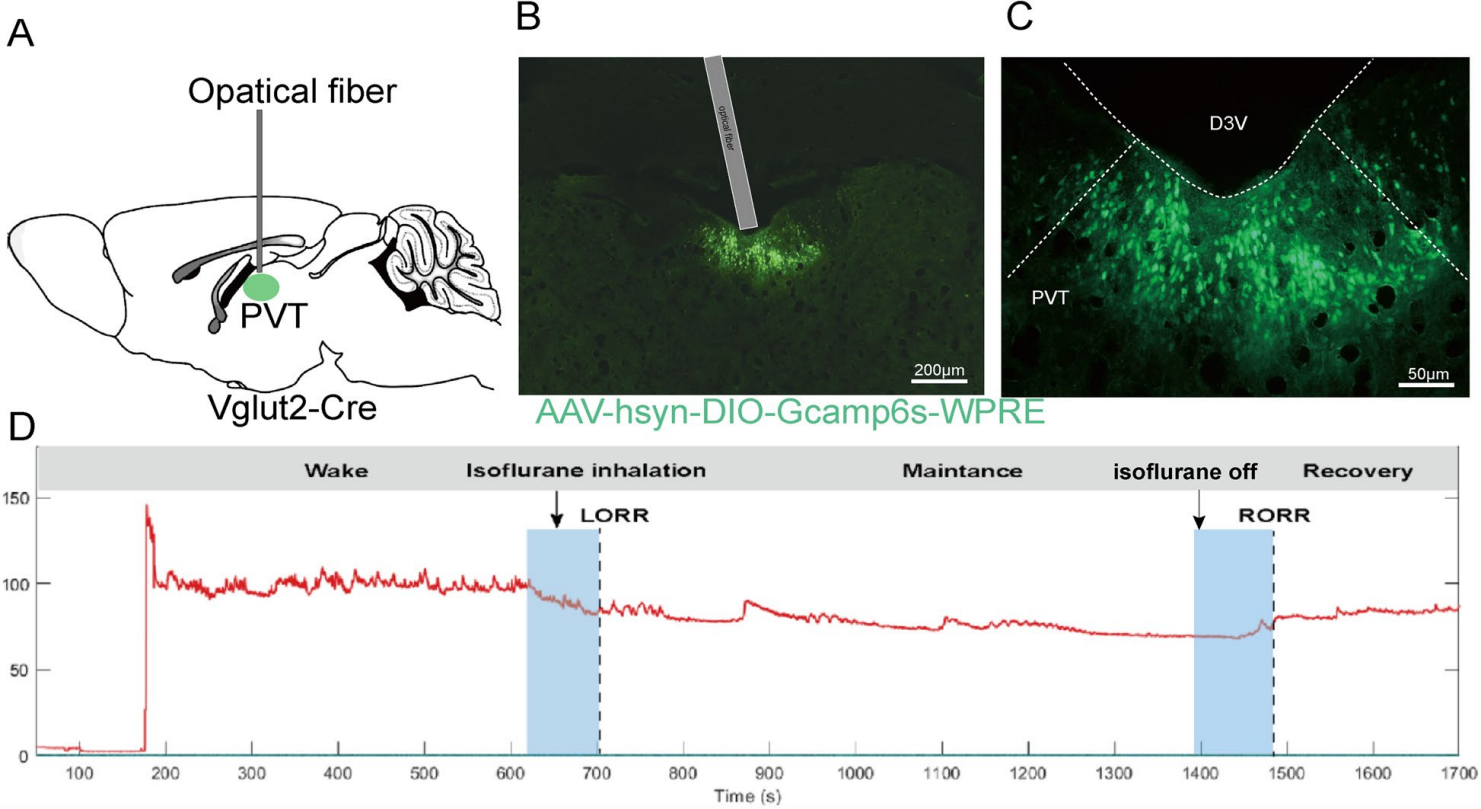
Phase-dependent calcium alterations in PVT neurons during isoflurane anesthesia. **A** Schematic of the AAV-hSyn-DIO-Gcamp6s-WPRE virus’ site. **B** Histological immunohistochemical photograph showing the AAV-hSyn-DIO-Gcamp6s-WPRE virus vector and fiber injecting sites in the PVT. (scale bar = 200 μm). **C** Higher magnification photograph of (**B**) (scale bar = 50 μm). **D** Real time recording of calcium signals in different states

To observe the real-time changes of the glutamatergic neurons in PVT during isoflurane anesthesia, we injected Cre-dependent AAV-hSyn-DIO-Gcamp6s into PVT neurons of Vglut2-IRES-Cre mice (Fig. 2A) and used fiber photometry to record changes in Ca^2+^ signals in vivo during isoflurane anesthesia (Figs. 2B and C).

**Fig. 3:**
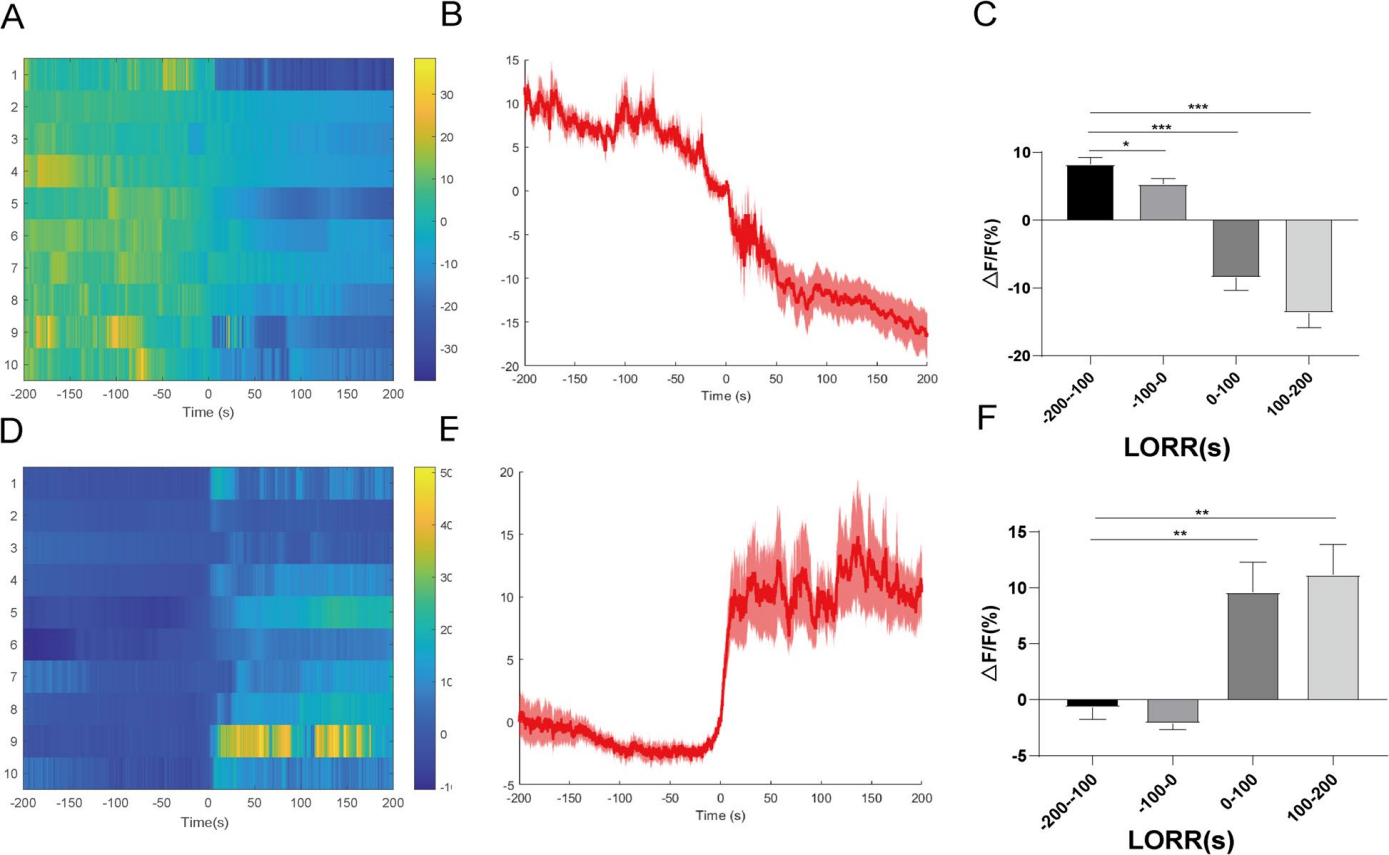
Neural dynamics of PVT in response to isoflurane. **A** Fluorescence calcium signals aligned to isoflurane-induced loss of righting reflex (LORR). **B** ΔF/F represents change in GCaMPs fluorescence from the mean level before the isoflurane is given. Mean (red trace) ± SEM (shading) indicating the average calcium transients during isoflurane-induced LORR. **C** Statistical chart of changes in Ca^2+^ signals in isoflurane -induced LORR. **D** Fluorescence calcium signals corresponded to isoflurane-induced recovery of righting reflex (RORR). **E** Mean (red trace) ± SEM (shading) indicating the average calcium transients during RORR. **F** Statistical chart of changes in Ca^2+^ signals during RORR. ^*^P < 0.05, ^**^P < 0.01, *n* = 10

During isoflurane anesthesia, we analyzed Ca^2+^ signals in four sections: anesthesia induction period (- 200-100 s), anesthesia maintenance period (- 100-0 s), early recovery period (0-100 s), complete recovery period (100-200s, Fig. 2D). During the disappearance of righting reflex in mice, the calcium signal of PVT neurons was significantly weaker than that induced (Figs. 3A-C). In the recovery period of righting reflex in mice, the calcium signal of PVT neurons recovered significantly (Figs. 3D-F).

### Chemogenetic activation of glutamatergic neurons in PVT promoted recovery from isoflurane anesthesia

**Fig. 4:**
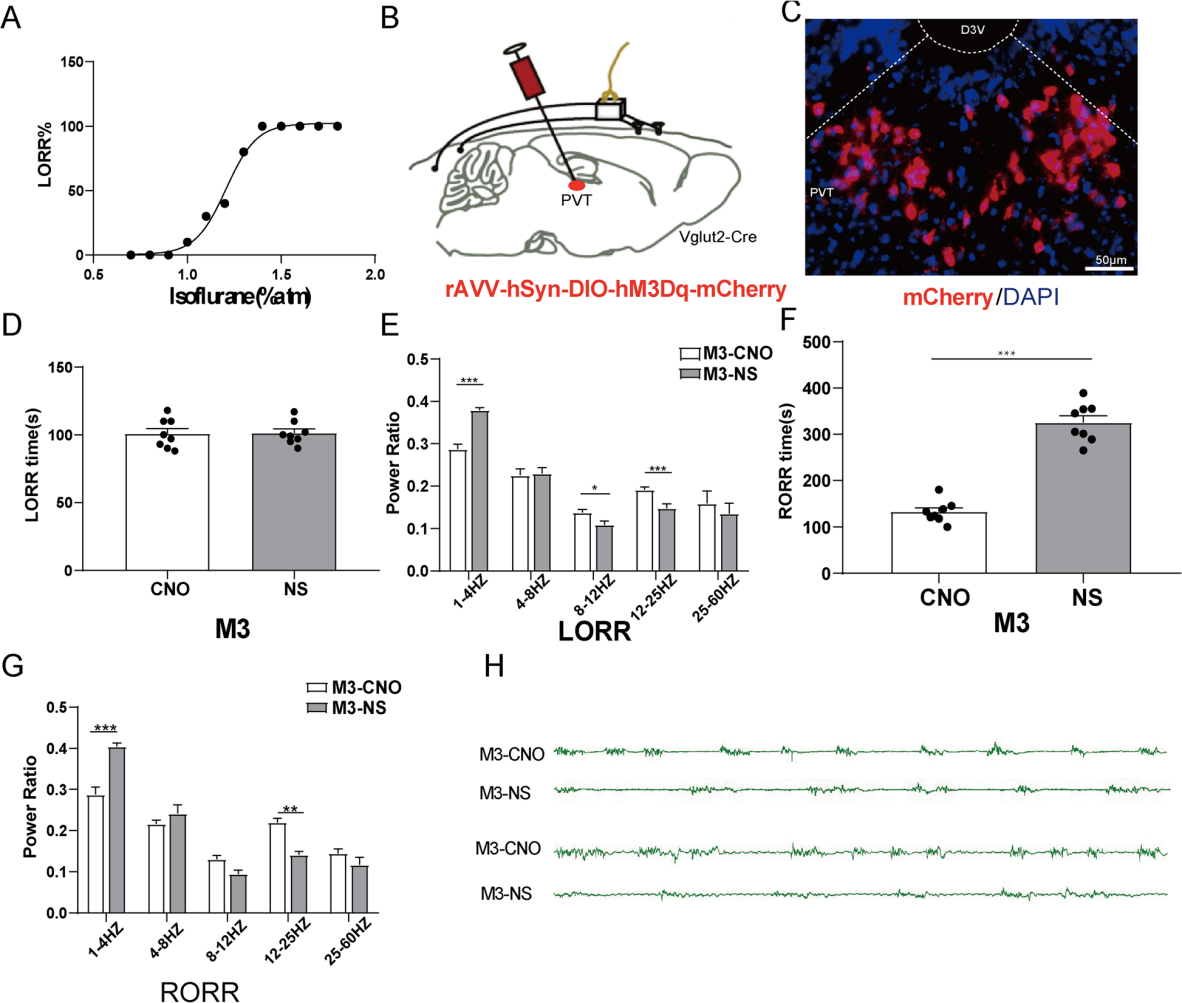
PVT glutamatergic neurons activation accelerates emergence from isoflurane anesthesia. **A** Relationship between the isoflurane concentration and % LORR. 1.4% isoflurane resulted in 100% LORR in mice. **B** Schematic of chemogenetic stimulation of glutamatergic neurons with EEG recordings. **C** Image of mCherry-expressing in PVT glutamatergic neurons (scale bar, 50 mm). **D** Effect of activation of PVT glutamatergic neurons on LORR time. **E** Effect of activation of PVT glutamatergic neurons on Cortical EEG during LORR. **F** Effect of activation of PVT glutamatergic neurons on RORR time. **G** Effect of activation of PVT glutamatergic neurons on Cortical EEG during RORR. **H** Representative EEG traces.^*^P < 0.05, ^**^P < 0.01, ^***^P < 0.001, *n* = 8

Before the experiments, we drew the dose effect curve of isoflurane, as shown in Fig. 4A. the isoflurane concentrations of 0.7%, 0.8%, 0.9%, 1.0%, 1.1%, 1.2%, 1.3%, 1.4%, 1.5%, 1.6%, 1.7% and 1.8% were measured respectively. It was found that the isoflurane concentration of 1.4% was the lowest inhalation concentration that could anesthetize mice 100% (*n* = 10), Therefore, 1.4% isoflurane was used in subsequent tests (Fig. 4A). To specifically activate glutamatergic neurons, we injected rAVV-hSyn-DIO-hM3Dq- mCherry into the PVT of Vglut2-Cre mice (Fig. 4B). Immunofluorescence images validated the virus transfection in PVT glutamatergic neurons (Fig. 4C).

In terms of behavior, as shown in Fig. 4D and 4F, the LORR time of M3^CNO^group was 100.75 ± 3.83 s and that of M3^NS^ group was 101.25 ± 3.03 s. There was no significant difference between the two groups (Fig. 4D, *P* > 0.05). The RORR time of M3^CNO^ group was 132.38 ± 8.36 s and that of M3^NS^ group was 325.38 ± 14.70 (Fig. 4F, *P* < 0.001). Hence, chemogenetic activation of glutamatergic neurons in PVT had no effect on the induction of isoflurane anesthesia, but accelerated the recovery from isoflurane anesthesia.

Cortical EEG (Fig. 4H), during LORR, the power of δ waves in M3^CNO^group was lower than that in M3^NS^ group (*P* < 0.001), α waves and β waves in M3^CNO^group were higher than that in M3^NS^ group (*P* < 0.05, Fig. 4E). During RORR, the power of δ waves in M3^CNO^group was also lower than that in M3^NS^ group (*P* < 0.001) and β waves in M3^CNO^group was also higher than that in M3^NS^ group (*P* < 0.01, Fig. 4G). These results suggest that specific activation of PVT glutamatergic neurons causes cortical EEG arousal.

### Chemogenetic inhibition of glutamatergic neurons in PVT prolonged recovery from isoflurane anesthesia

**Fig. 5:**
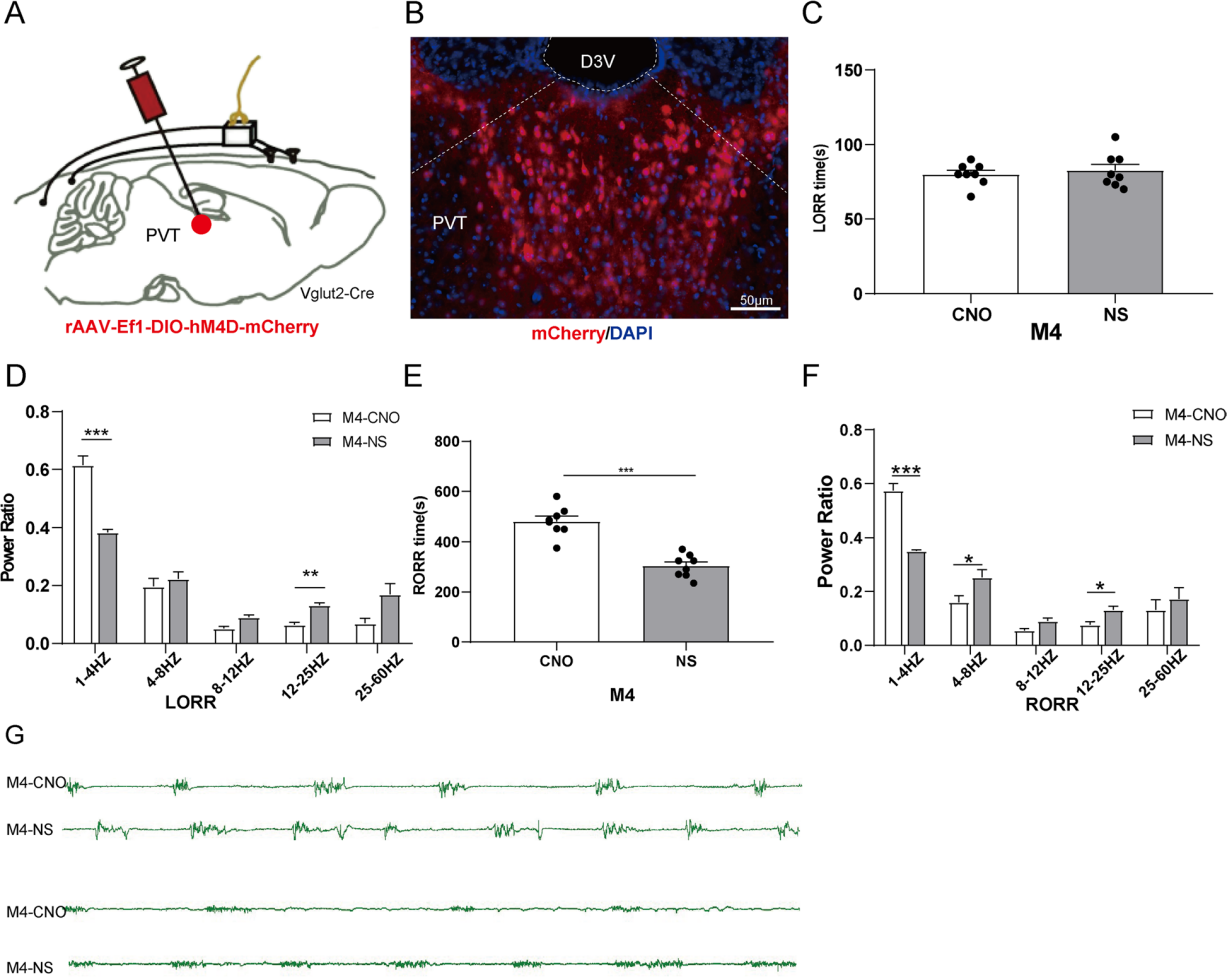
PVT glutamatergic neurons inhibition delays emergence from isoflurane anesthesia. **A** Schematic of chemogenetic stimulation of glutamatergic neurons with EEG recordings. **B** Image of mCherry-expressing in PVT glutamatergic neurons (scale bar, 50 mm). **C** Effect of inhibition of PVT glutamatergic neurons on LORR time. **D** Effect of inhibition of PVT glutamatergic neurons on Cortical EEG during LORR. **E** Effect of inhibition of PVT glutamatergic neurons on RORR time. **F** Effect of inhibition of PVT glutamatergic neurons on Cortical EEG during RORR. **G** Representative EEG traces.^*^P < 0.05, ^**^P < 0.01, ^***^P < 0.001, *n* = 8

To specifically inactivate glutamatergic neurons, we injected rAVV-Ef1-DIO-hM4D-mCherry into the PVT of Vglut2-Cre mice (Fig. 5A). Immunofluorescence images validated the virus transfection in PVT glutamatergic neurons (Fig. 5B). The LORR time of M4^CNO^ group was 80 ± 2.67 s and that of M4^NS^ group was 82.63 ± 4.11 s. There was no significant difference between the two groups (Fig. 5C, *P* > 0.05). The RORR time of M4^CNO^ group was longer than that of M4^NS^ group (481 ± 21.15 s *vs* 303.38 ± 16.08 s, *P* < 0.001, Fig. 5E).

Cortical EEG (Fig. 5G), during LORR, the power of δ waves in M4^CNO^ group was significantly higher than that in M4^NS^ group and the power of β waves was lower than that in M4^NS^ group (*P* < 0.001, Fig. 5D). Compared with the M4^NS^ group, the δ waves in the M4^NS^ group increased significantly during RORR (*P* < 0.001), and _θ_ wave and β waves decreased (*P* < 0.05, Fig. 5F). These results indicate that specific inhibition of PVT-Gluergic neurons has no effect on the induction time of isoflurane anesthesia, but can inhibit cortical awakening; prolong the emergency from anesthesia and inhibit cortical awakening at the same time.

## Discussion

In the past few decades, PVT has been considered as an important node of limbic system, which is related to emotional processing [12, 13]. A recent study demonstrated that PVT is also a key wakefulness-controlling nucleus in the thalamus [14]. Our results show that PVT glutamatergic neurons are involved in isoflurane anesthesia.

Some previous studies have demonstrated that some brain regions regulate recovery form anesthesia [10, 11, 15–17]. Our c-Fos immunostaining data shows that PVT neurons are inactive during isoflurane anesthesia and the activity partial restores after emergence from isoflurane anesthesia. A recent study reveals that locus coeruleus (LC), tyrosine hydroxylase (TH) neurons are active during passive recovery from isoflurane anesthesia [18]. Ao et al. also found that the activity of PVT neurons is enhanced after recovery from isoflurane anesthesia [19]. In conclusion, PVT neurons are involved in isoflurane anesthesia.

In addition to c-Fos protein, calcium fiber photometry is another sensitive method to record neuronal activities in living animals [20]. This technique can record the activity of neurons in real time [21–23]. Glutamatergic neurons were dominant in PVT [13]. In our study, the changes of Ca^2+^ signals of glutamatergic neurons in PVT were recorded in real time. We found that the population Ca^2+^ activity of PVT glutamatergic neurons decreased during isoflurane anesthesia and increased during emergence. Similarly, the population Ca^2+^ signals of PVT neurons were significantly higher during wakefulness than during sleep [14]. Therefore, PVT glutamatergic neurons are involved in isoflurane anesthesia.

In order to further clarify the effect of PVT glutamatergic neurons on isoflurane anesthesia, we used chemogenetic method to precisely regulate PVT glutamatergic neurons. The chemogenetic strategy has been successfully applied in the activation of PVT glutamatergic neurons in wakefulness-controlling study [8]. In our study, the activation of glutamatergic neurons in PVT did not affect the induction time of isoflurane anesthesia, but accelerated the recovery of anesthesia. On the contrary, inhibition of PVT neurons also did not affect the induction time of anesthesia and prolonged the recovery time of isoflurane anesthesia. These results suggest that PVT glutamatergic neurons are not involved in the process of anesthesia induction, but in the emergence process of isoflurane anesthesia. A sleep-arousal study also found that the inhibition of PVT neuronal activity induced a reduction in wakefulness, whereas activation of PVT neurons induced a transition from sleep to wakefulness [8]. Therefore, PVT is the key nucleus of thalamus regulating wakefulness.

In addition to behavioral changes, activation or supression of PVT glutamatergic neurons also caused changes in cortical EEG. In this study, chemogenetically activation of PVT glutamatergic neurons decreased the power of δ waves, increased the the power of α waves and β waves. On the one hand, when the PVT glutamatergic neurons were chemogenetically inhibited, a high EEG δ power (2–4 Hz) increased markedly. The lesion of PVT glutamatergic neurons with diphtheria toxin A or ibotenic acid equally induced a decrease in wakefulness and an increase in EEG δ power [8]. When PVT glutamatergic neurons were optogenetically activated, a decrease in EEG δ power occurred [8]. These data indicates that the PVT is both necessary and sufficient for wakefulness.

The study has some limitations. Firstly, respiration and heart rate are not recorded. Increased alveolar minute ventilation and increased cardiac output, both of which may have occurred with PVT neuron stimulation, alter isoflurane distribution and elimination rate in the body and can decrease alveolar isoflurane partial pressure and accelerate awakening from isoflurane anaesthesia. In this study, we didn’t monitor heart rate and respiratory rate in the M3^NS^ and M3^CNO^ groups after CNO and NS were administered, respectively. Similarly, heart rate and respiratory rate were not monitored in the M4^NS^ and M4^CNO^ groups. Although the doses of CNO and NS applied didn’t affect the behavior and cortical EEG of the mice in control group [9], the effect of activation PVT promoting anesthesia recovery still cannot exclude pharmacokinetic explanation for the results observed in the present study. Secondly, although we found that PVT glutamatergic neurons are involved in the regulation of isoflurane anesthesia recovery, it is still unclear whether other types of neurons are also involved. Finally, although we found that PVT glutamatergic neurons are involved in the regulation of isoflurane anesthesia awakening, which pathway they participate in the regulation of wakefulness is still unknown.

## Conclusions

Assuming a pharmacokinetic explanation for results can be excluded, PVT was involved in the regulation of isoflurane anesthesia recovery, activation of PVT promoted anesthesia recovery, inhibition of PVT delayed anesthesia recovery, but had no effect on anesthesia induction.

## Data Availability

The datasets generated and analyzed during the current study are available from the corresponding author on reasonable request.

## Abbreviations

PVT: Paraventricular nucleus of the thalamus
EEG: Electroencephalography
CNO: Clozapine N-oxide
NS: Normal saline
LORR: Loss of righting reflex
RORR: Recovery of righting reflex
PBS: Phosphate-bufered saline
LC: Locus coeruleus
TH: Tyrosine hydroxylase
